# Gallbladder cancer masquerading as xanthogranulomatous cholecystitis: a case report and literature review

**DOI:** 10.3389/fonc.2024.1409347

**Published:** 2024-07-17

**Authors:** Xu Deng, Chun-yuan Yang, Wei Tian, Zong-long Zhu, Jian-xing Tian, Rui Huang, Ming Xia, Wei Pan

**Affiliations:** Department of Hepatobiliary and Pancreatic Surgery, the People’s Hospital of Lezhi, Ziyang, China

**Keywords:** xanthogranulomatous cholecystitis (XGC), gallbladder cancer (GBC), laparoscopic cholecystectomy (LC), Surgery 5, pathology

## Abstract

Xanthogranulomatous cholecystitis (XGC) is a rare type of cholecystitis that, despite being benign poses diagnostic challenges due to its low prevalence and need for consensus on diagnostic criteria. Consequently, distinguishing XGC from gallbladder cancer (GBC) is challenging, leading to clinical misdiagnoses. This article presents a case where a patient initially diagnosed with GBC was later found to have XGC.

## Introduction

Xanthogranulomatous cholecystitis (XGC), once mistaken for a malignant disease, is now recognized as benign condition (1). It is characterized by atypical thickening of the gallbladder wall and infiltration of yellow granulomatous tissue, occasionally invading surrounding organs such as the liver, duodenum, colon, and common bile duct (2). Previous studies indicate XGC prevalence ranges from 1.3% to 1.9%, predominantly affecting individuals aged 60–70 (3, 4). Distinguishing XGC from gallbladder cancer (GBC) by conventional imaging is challenging, and even intraoperative frozen sections can yield false negatives. In this article, we present a case where both preoperative and intraoperative frozen sections were positive for XGC, whereas postoperative paraffin sections indicated presence of GBC.

## Case report

An 80-year-old Chinese male was hospitalized due to recurring epigastric pain and discomfort lasting over for three years, with recent exacerbation for a week before admission.

More than three years ago, he experienced subxiphoid pain with nausea, dry heaving, and radiating back pain. An abdominal ultrasound conducted at a local hospital indicated gallbladder stones of unknown size. He opted against surgery and was discharged after receiving symptomatic supportive therapy to alleviate the symptoms. A week before admission to our hospital, he reported intolerable epigastric pain, and the medical staff recommended surgical intervention. Aside from a decade-long history of chronic bronchitis with emphysema, the patients had no history of chronic diseases such as hypertension and diabetes mellitus. In addition, he had no history of smoking, alcoholism, specific hereditary diseases, and prior surgery.

**Figure 1:**
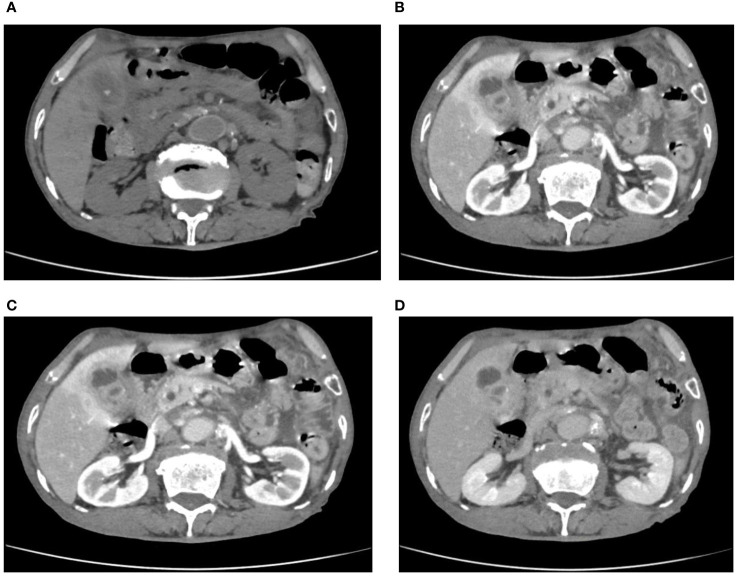
**(A)** calculus within the gallbladder and common bile duct were seen on CT plains; **(B–D)** enhanced CT showed extensive inhomogeneous thickening of the gallbladder wall with intramural hypodensity.

After admission, an ultrasound examination revealed significant gallbladder wall thickening with strong echoes visible in the capsule, accompanied by a posterior acoustic shadow. A computed tomography (CT) scan was conducted to examine suspected cholecystitis and choledocholithiasis associated with gallbladder stones. An enhanced CT scan indicated liver contrast abnormalities, suggesting a diagnosis of XGC, but not ruling out GBC (**Figure 1**). Further analysis showed that the relevant tumor marker levels were CA-125 level at 239 U/ml, CA19–9 less than 2.0 U/ml, and the levels of inflammatory markers were CRP at 36.95 mg/L, WBC at 6.26×10^9/L, and % neutrophil at 66.00%. Despite these findings, the possibility of malignancy could not be excluded. After discussing the situation with his family, they agreed to proceed with a surgical procedure adjusted based on the results of the intraoperative frozen section biopsy.

**Figure 2:**
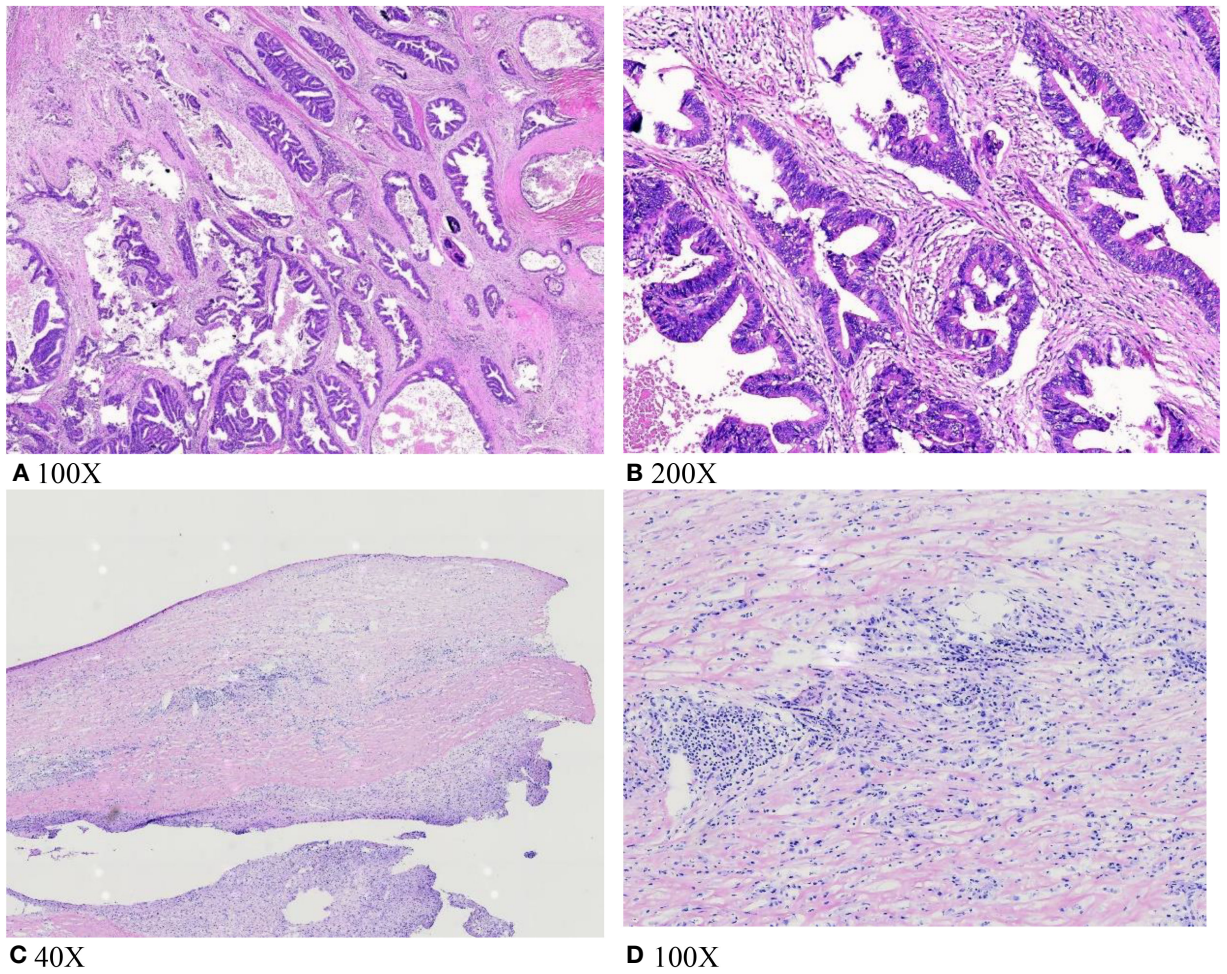
**(A, B)** Tumor cell infiltration was seen on postoperative pathological paraffin sections; **(C, D)** intraoperative frozen section biopsy did not show tumor cell infiltration; foam cell infiltration was seen.

During surgery, we took a small tissue sample suspected to be a tumor and conducted intraoperative freezing, as the gallbladder was significantly inflamed and poorly defined from the liver tissue. This feature prevented complete separation of the gallbladder from the gallbladder bed. Intraoperative pathology revealed no tumor cells, but foam cell infiltration was observed (**Figure 2**). Consequently, the patient underwent a 6-hour-long procedure, comprising laparoscopic partial hepatectomy, cholecystectomy, choledochotomy for lithotripsy, and T-tube drainage, without lymph node dissection. Intraoperative bleeding was approximately 300 ml. Postoperatively, he recovered well without significant complications. However, the postoperative paraffin section pathology indicated adenocarcinoma of the gallbladder (**Figure 2**). After receiving these results, the family opted against further surgical treatment due to the patient’s age. The patient recovered and was discharged.

## Discussion

XGC is a distinct form of cholecystitis characterized by localized or widespread inflammatory changes (5). For the vast majority of patients with cholecystitis, the progression is as follows: in the first 2 to 4 days, there is marked congestion and oedema, in 3 to 5 days, necrotising cholecystitis occurs, from the 7th to 10th day, the disease enters a suppurative phase, and after 2 to 3 weeks, the suppurative foci are replaced by granulation tissue, which gradually progresses to subacute cholecystitis and eventually to chronic cholecystitis (6). Macroscopically, the gallbladder wall is thick with a solid mass or yellow-brown nodules. Microscopically, it exhibits infiltration of (i) foamy macrophages or bile-containing macrophages, (ii) focal, nodular or diffuse fibrotic proliferation; and (iii) significant infiltration of inflammatory cells such as lymphocytes, plasma cells, foreign body giant cells and neutrophils (7). The formation of yellow-brown nodules is attributed to increased pressure within the gallbladder caused by biliary obstruction or cholecystitis, ultimately leading to mucosal damage and bile entry into the gallbladder wall. The bile is phagocytosed by foam cells or macrophages and forms a tumor-like mass (3). Within some cases, XGC can invade adjacent organs due to its destructive inflammatory nature, resembling the infiltrative growth observed in tumors (8). Therefore, distinguishing XGC from GBC can be clinically challenging.

XGC and GBC have similar clinical manifestations. Patients with the two conditions present with pain and discomfort in the right upper abdomen or subxiphoid region, and symptoms such as obstructive jaundice, and an enlarged gallbladder (7). Laboratory indicators do not clearly differentiate these conditions. Tumor markers such as CEA and CA19–9 lack specificity as they can be elevated or within the normal ranges in XGC and GBC patients. Elevated CA19–9 in XGC may be caused by inflammation-induced bile duct damage, resulting in increased secretion of CA19–9 by epithelial cells (9). Conversely, some GBC patients may have normal tumor marker levels. In the present case, CA19–9 levels were not elevated. However, Kha et al. reported that tumor markers can be used for postoperative follow-up monitoring (10). The levels of tumor markers in XGC patients may decrease after surgery, whereas they remain elevated in GBC patients, offering a relatively reliable means of identification. And in a recent study it was noted that IgG4-related disease (IgG4-RD) is an emerging and recently recognized disease entity that can affect virtually all the organs and can have myriad manifestations. The disease is associated with elevated levels of serum IgG4, and is characteristically responsive to steroids. Checking IgG4 levels in patients with suspected xantogranulomatous cholecystitis in the preoperative period may be useful in supporting the preoperative diagnosis (11). Currently, imaging is the most reliable approach for accurate diagnosis.

Ultrasound is commonly chosen for clinical evaluation due to its non-invasive and convenient nature (4). Gupta et al. established the Gallbladder reporting and data system (GB-RADS) to aid in distinguishing between benign and malignant diseases (11). This system standardizes common terminology to describe the gallbladder lumen and wall characteristics in ultrasound images. However, GB-RADS does not apply to acute cholecystitis or other non-cystic causes of gallbladder wall thickening (11). Cui et al. reported delamination associated with fat-rich macrophages (or foam cells) and severe fibrosis is observed in some XGC patients (2). Despite these insights, Doppler flow ultrasound has low efficacy in differentiating between XGC and GBC due to neovascularization in the two conditions (4). CEUS has emerged as a more effective tool in clinical practice, offering superior detection of gallbladder wall thickness and hypoechoic nodules compared to conventional ultrasound, potentially aiding in differentiation of XGC from GBC. However, the specificity of ultrasound in diagnosing XGC is relatively low.

Therefore, further CT or MRI examination is required when ultrasound reveals nodular-like changes within the gallbladder. Several studies report that the characteristics of XGC include (i) diffuse or localized thickening of the gallbladder wall, (ii) hypodense nodules visible in the capsule, and (iii) intact and continuous gallbladder wall mucosa (3, 4, 8). Conversely, GBC often presents with limited gallbladder wall thickening and disrupted mucosal integrity (8). Xiao et al. observed that the hypodense nodules surrounding the affected area aid in distinguishing XGC from GBC (7). Combined CT and MRI imaging features are valuable in diagnosing XGC. Zhou et al. constructed a diagnostic prediction model incorporating 11 imaging features, achieving an AUC of 0.888 and an accuracy of 0.898. These features include T2WI signal of intramural nodules, T1WI signal of intramural nodules, lipid signal, gallstones, mucosal lines, apparent diffusion coefficient (ADC), peripheral lymph nodes, DWI, T2WI signal of thickened cyst wall, bile duct dilation, and intramural nodules (3). In a multiparametric MRI study, subgroup analysis comparing patients with XGC and GBC revealed that heterogeneous enhancement of the gallbladder wall was significantly associated with GBC. Furthermore, quantitative MRI parameters indicated a tendency for higher MD and TTP in XGC compared to GBC (12). ^18^F-FDG PET/CT is a valuable imaging approach for detecting malignant gall bladder lesions (13). However, its high SUV in inflammatory diseases compromises its accuracy in differentiating between benign and malignant pathologies (13). ^18^F-fluorothymidine (FL-T) overcomes this imaging limitation and is extensively studied as an imaging agent for assessing tumor cell proliferation. The diagnostic accuracy of FL-T-PET/CT in differentiating benign and malignant biliary tumors is 92%, which is superior to the accuracy of FDG-PET/CT and CECT methods (14).

In addition to conventional imaging, the application of deep-learning (DL) models on ultrasound (US) images has demonstrated diagnostic accuracy comparable to that of radiologists in distinguishing between XGC and GBC (15, 16). This approach represents a promising area for future research.

However, pathology remains the gold standard for diagnosis of these conditions. Fine-needle aspiration biopsy or an intraoperative frozen section can aid in diagnosis (8, 17). However, false negatives are caused by several factors. XGC and GBC coexist in 2–15% cases (10), and technical limitations may prevent sampling of lesions in some patient. In the present case, complete gallbladder removal without compromising its integrity was challenging due to extensive inflammatory response. Therefore, tissue samples suspected of tumor lesions were obtained and sent for frozen section biopsy. Despite observing foam cell infiltration in these samples, no tumor cells were identified, leading to a diagnosis of XGC.

Once XGC is diagnosed, laparoscopic cholecystectomy (LC) is the preferred treatment option. This is primarily because XGC often exhibits infiltrative inflammation that can extend into the surrounding tissues and cause inflammatory rupture (18). XGC patients have a higher rate of surgical complications compared to patients with typical cholecystitis (13.5–43.5% vs. 2.6%), including complications such as biliary fistulae due to damage to bile ducts in poorly demarcated gallbladder triangles, or pleural effusions (9). In some cases, patients require intermediate laparotomy due to severe inflammatory infiltration, unclear tissue structure, and strong adhesion to surrounding tissues. Kim et al. reported a 10–80% rate of midline laparotomy in these cases (19). If XGC or GBC cannot be effectively differentiated preoperatively, the surgical approach can vary. GBC patients (except for stage 0–1 patients) often require radical cholecystectomy, which is associated with higher surgical risks and more postoperative complications than conventional LC. In clinical practice, some GBC cases are incidentally discovered after surgery. In China, there is still an opportunity to perform radical surgery within 1–4 weeks after surgery for these patients. In the case presented in this study, when the patient was informed about the possibility of a second surgical procedure within 1–4 weeks, the patient and his family opted against the radical surgical approach. This decision is unfortunate because GBC patients generally have poor prognosis.

## Conclusion

In summary, preoperative imaging aids in distinguishing between XGC and GBC. However, accurately differentiating XGC from GBC is challenging. Therefore, preoperative fine-needle aspiration or intraoperative frozen biopsy is essential for accurate diagnosis. Conducting multiple frozen section biopsies intraoperatively helps minimize the risk of false negatives. Further research and development of new diagnostic modalities are anticipated to improve the differentiation between these two diseases.

## Data availability statement

The raw data supporting the conclusions of this article will be made available by the authors, without undue reservation.

## Ethics statement

The studies involving humans were approved by the People’s Hospital of Lezhi. The studies were conducted in accordance with the local legislation and institutional requirements. The participants provided their written informed consent to participate in this study. Written informed consent was obtained from the individual(s) for the publication of any potentially identifiable images or data included in this article.

## Author contributions

XD: Writing – original draft, Writing – review & editing. CY: Writing – original draft, Writing – review & editing. WT: Writing – review & editing. ZZ: Writing – review & editing. JT: Writing – review & editing. RH: Writing – review & editing. MX: Writing – review & editing. WP: Writing – original draft, Writing – review & editing.
